# Prácticas de fin de vida y directivas anticipadas de voluntad: un análisis bioético a partir de las discusiones en el Poder Legislativo Federal brasileño

**DOI:** 10.18294/sc.2026.5939

**Published:** 2026-03-11

**Authors:** Melisse Eich, Marta Verdi, Pedro Paulo Scremin Martins, Mirelle Finkler

**Affiliations:** 1 Posdoctoranda, Programa de Pós-graduação em Saúde Coletiva, Universidade Federal de Santa Catarina. Professora voluntáriado, Departamento de Saúde Pública, Universidade Federal de Santa Catarina. Membro e pesquisadora, Núcleo de Pesquisa e Extensão em Bioética e Saúde Coletiva, Santa Catarina, Brasil. meliseeich@hotmail.com Universidade Federal de Santa Catarina Departamento de Saúde Pública Universidade Federal de Santa Catarina Santa Catarina Brazil meliseeich@hotmail.com; 2 Doctora en Enfermería, Professora adjunta, Departamento de Saúde Pública; professora permanente, Programa de Pós-Graduação em Saúde Coletiva, Universidade Federal de Santa Catarina. Líder, Núcleo de Pesquisa e Extensão em Bioética e Saúde Coletiva, Santa Catarina, Brasil. verdiufsc@gmail.com Universidade Federal de Santa Catarina Departamento de Saúde Pública Programa de Pós-Graduação em Saúde Coletiva Universidade Federal de Santa Catarina Santa Catarina Brazil verdiufsc@gmail.com; 3 Magíster en Filosofía. Estudiante, Doctorado em Saúde Coletiva, Universidade Federal de Santa Catarina, Santa Catarina, Brasil. ppsm29@hotmail.com Universidade Federal de Santa Catarina Universidade Federal de Santa Catarina Santa Catarina Brazil ppsm29@hotmail.com; 4 Doctora en Odontología. Profesora asociada, Departamento de Odontologia; profesora permanente, Programa de Pós-Graduação em Saúde Coletiva, Universidade Federal de Santa Catarina. Vicelíder, Núcleo de Pesquisa e Extensão em Bioética e Saúde Coletiva, Santa Catarina, Brasil. mirellefinkler@yahoo.com.br Universidade Federal de Santa Catarina Departamento de Odontologia Programa de Pós-Graduação em Saúde Coletiva Universidade Federal de Santa Catarina Santa Catarina Brazil mirellefinkler@yahoo.com.br

**Keywords:** Cuidados Paliativos, Directivas Anticipadas, Autonomía Personal, Derecho a Morir, Eutanasia, Brasil, Palliative Care, Advance Directives, Personal Autonomy, Right to Die, Euthanasia, Brazil

## Abstract

El debate legislativo brasileño sobre prácticas de fin de vida involucra tensiones ético-axiológicas relacionadas con la eutanasia, el suicidio asistido, los cuidados paliativos y las directivas anticipadas de voluntad, en el que se confrontan concepciones divergentes de estas prácticas y el valor de la vida, influyendo en la formulación normativa del proceso de morir. Este estudio analiza cómo tales tensiones son construidas y justificadas en los discursos del Poder Legislativo Federal, comprendiendo los sentidos normativos y la jerarquía de valores atribuidos a los cuidados paliativos y a las directivas anticipadas de voluntad, así como sus implicaciones ético-morales. Se realizó una investigación documental cualitativa, orientada por la hermenéutica-dialéctica y fundamentada en referentes contemporáneos de la bioética de la responsabilidad y de la bioética cotidiana. Se examinaron 193 documentos legislativos federales (1981-2020). Del análisis emergieron dos categorías temáticas: 1) *conjunto paliativo: sentidos y atribuciones*, que evidencia la tendencia legislativa a limitar el derecho a la negativa terapéutica; y 2) *instrumentos y mecanismos de acción para la inversión de las directivas anticipadas de voluntad*, que revela la restricción de instrumentos destinados a la protección de la autonomía. Los resultados señalan la prevalencia de argumentos basados en valores vitales, tratados como absolutos, en detrimento de valores personales como la dignidad humana, la autonomía y la autodeterminación, lo que puede restringir el derecho a morir con dignidad en Brasil.

## Abstract (en)

The Brazilian legislative debate on end-of-life practices involves ethical-axiological tensions related to euthanasia, assisted suicide, palliative care, and advance directives, in which divergent conceptions of these practices and of the value of life confront one another, influencing the normative formulation of the dying process. This study analyzes how such tensions are constructed and justified in the discourses of the Federal Legislative Branch, examining the normative meanings and the hierarchy of values attributed to the palliative care framework and to advance directives, as well as their ethical and moral implications. A qualitative documentary study was conducted, guided by a hermeneutic-dialectical approach and grounded in contemporary frameworks of the bioethics of responsibility and everyday bioethics. A total of 193 federal legislative documents (1981-2020) were examined. The analysis yielded two thematic categories: (1) *the palliative care framework: meanings and attributions*, which reveals the legislative tendency to limit the right to therapeutic refusal; and (2) *instruments and mechanisms for the reversal of advance directives*, which exposes restrictions on instruments intended to protect autonomy. The findings indicate the predominance of arguments grounded in vital values treated as absolute, to the detriment of personal values such as human dignity, autonomy, and self-determination, which may restrict the right to die with dignity in Brazil.

## Introducción

Las concepciones del mundo y las visiones morales sobre el proceso de vivir y morir confluyen en la comprensión de que los otros son respetables no por compartir creencias semejantes, sino, precisamente, por ser otros, otros seres humanos, otros como yo. Esta premisa expresa la universalidad de la condición humana y fundamenta la idea de que los cuidados paliativos abarcan el proceso de morir y la muerte y, por lo tanto, están permeados por valores éticos centrales, entre los cuales se destaca la dignidad de la persona humana.

A partir de esta relación entre universalidad de los principios y particularidad de los valores, se vuelve necesario reflexionar sobre los conflictos inherentes a la vida y sobre los valores socialmente compartidos. No se deben confundir los valores con sus soportes materiales. Los seres humanos se constituyen en un movimiento dinámico de aprehensión de la realidad, y tanto sus valores como sus soportes se transforman a lo largo de la vida. En este recorrido, se acumulan experiencias y responsabilidades que moldean respuestas éticas frente a los obstáculos y demandas sociales, en un proceso continuo de interacción entre factores biológicos, psicológicos, culturales e históricos. Así, elementos esenciales que componen al ser social y orientan el vivir y el morir se articulan con la dignidad personal, construida históricamente en el movimiento dinámico de la vida cotidiana e integrada a la propia biografía de cada persona^1^^,^^2^^,^^3^.

Es en este contexto de tensión entre lo universal y lo particular que las prácticas de fin de vida -como la eutanasia voluntaria activa, el suicidio asistido, la ortotanasia y las directivas anticipadas de voluntad- se han convertido en objeto de disputas éticas, normativas y políticas en Brasil, especialmente en el ámbito del Poder Legislativo Federal. En las últimas cuatro décadas, proyectos de ley, dictámenes y discursos parlamentarios han movilizado valores morales, concepciones divergentes de dignidad humana e interpretaciones distintas sobre autonomía, responsabilidad y sufrimiento. Este proceso produjo tensiones internas, contradicciones normativas y distorsiones conceptuales sobre prácticas consolidadas en los cuidados paliativos, tales como la limitación de tratamientos fútiles, la sedación paliativa y las directivas anticipadas de voluntad.

De este proceso emerge una contradicción normativa central que justifica esta investigación. Aunque la Constitución de la República Federativa de Brasil (CRFB) de 1988 consagra la dignidad de la persona humana como principio fundamental (art. 1º, III) y reconoce la autonomía individual como pilar esencial para su plena realización^4^, el Poder Legislativo brasileño ha presentado múltiples proposiciones que criminalizan o restringen prácticas de fin de vida -incluyendo la eutanasia y el suicidio asistido, ya criminalizados desde el Código Penal de 1940- y, paradójicamente, ha reconocido acciones como éticamente legítimas en los cuidados paliativos, como el derecho a rechazar tratamientos fútiles^5^ y solicitar sedación paliativa^6^^,^^7^. Tales propuestas no solo restringen prácticas de fin de vida, sino que, de manera general, se contraponen a la dignidad de la persona humana, cuyo principio constitucional “reforzó la necesidad de considerar la voluntad del paciente y su derecho a elegir una muerte digna, sin la imposición de medidas terapéuticas desproporcionadas”^8^ y sin prolongar artificialmente la vida en el proceso de morir (ortotanasia)^9^.

Comprender cómo operan estos discursos es esencial para identificar de qué modo ciertas concepciones morales particulares se convierten en propuestas legales que pueden afectar directamente el proceso de morir en el país. Es en este contexto que se inscribe la pregunta de investigación que guía este estudio: ¿cómo los proyectos de ley y demás documentos producidos por el Poder Legislativo brasileño, entre 1981 y 2020, construyen sentidos morales y normativos para las prácticas de fin de vida (especialmente las directivas anticipadas de voluntad) y de qué manera estas construcciones expresan conflictos axiológicos, tensiones morales y jerarquías de valores que influyen en la autonomía, la dignidad y la responsabilidad moral en el proceso de morir? Este problema emerge de un contexto en el cual los discursos parlamentarios recurren a la defensa de valores vitales -situados en los niveles inferiores de la jerarquía axiológica, según Diego Gracia^2^- para sostener posiciones políticas que, predominantemente, no dialogan con el conocimiento técnico-científico, los fundamentos de los cuidados paliativos o los marcos éticos contemporáneos sobre vida, muerte y deliberación moral.

En la disputa en torno a esta contradicción ético-normativa, los discursos legislativos se materializan en consecuencias concretas para el sistema de salud brasileño y para la vida de las personas en situación de terminalidad. Se trata, por lo tanto, de una cuestión ética fundamental que refleja la tensión entre el deber constitucional de respeto a la dignidad humana y a la autonomía individual, y proyectos legislativos que absolutizan el valor de la vida biológica, situándolo jerárquicamente por encima de estas dimensiones esenciales de la existencia humana.

El debate contemporáneo sobre las prácticas de fin de vida, en este sentido, explicita conflictos éticos profundos relacionados con la dignidad humana, el valor de la vida y la autonomía, entendida tanto en su papel pasivo, vinculado al respeto por la voluntad de los otros para actuar según sus propios esquemas de valores, como en su papel activo, relacionado con la promoción de actitudes de autodeterminación^10^. Cuando se trasladan a la arena normativa, estos conflictos pasan a configurar disputas políticas, en las que las acciones políticas hegemónicas imponen una concepción única de “muerte digna”, centrada en la prolongación biológica de la vida, en detrimento de valores como la dignidad y la autodeterminación, más directamente vinculados a la construcción biográfica de la persona. Tal inversión de la jerarquía axiológica debilita la posibilidad de que las personas en el final de la vida ejerzan plenamente el derecho fundamental a la autonomía en decisiones que afectan profundamente su identidad y proyectos de vida, pues, como afirma Gracia^2^: “la deliberación exige autonomía”.

Este estudio tiene como objeto analizar las tensiones que se construyen y se justifican en las proposiciones y discursos del Poder Legislativo Federal, investigando los sentidos y atribuciones conferidos a las prácticas de fin de vida y a las directivas anticipadas de voluntad, así como sus implicaciones ético-morales. La relevancia de este análisis reside en el hecho de que las decisiones legislativas moldean no solo políticas públicas, sino también valores sociales sobre sufrimiento, autonomía, responsabilidad y dignidad humana al final de la vida. Además, una de las conclusiones analíticas del corpus es que los debates legislativos establecen disputas axiológicas que oponen valores asociados a la preservación de la vida biológica y valores personales vinculados a la libertad y la autonomía. Esto refuerza la necesidad de un análisis sistemático de los discursos parlamentarios y de los proyectos legislativos como espacio de producción normativa y de significación moral sobre las prácticas de fin de vida y las directivas anticipadas de voluntad. Estos debates, de hecho, aproximan al país a discusiones más amplias observadas en América Latina, las cuales cuestionan presupuestos del paradigma bioético hegemónico y fomentan una cultura crítica y libertaria^11^.

Para interpretar estas dinámicas, el estudio se fundamenta en dos marcos teóricos complementarios. La bioética de la responsabilidad de Diego Gracia propone que la condición humana se estructura en una jerarquía de valores en la que los valores vitales ocupan la base, mientras que los valores personales -como autonomía, dignidad, libertad, autodeterminación y realización personal- se sitúan en niveles superiores^2^^,^^3^^,^^12^. Gracia propone, además, una teoría de la deliberación ética entendida como un procedimiento universal de la racionalidad práctica, cuyo ámbito primario de aplicación son los valores (públicos y privados), que debe constituir la base de la deliberación política^2^. Este referente permite identificar cuándo las argumentaciones legislativas se apoyan en valores jerárquicamente inferiores -los “valores vitales”, frecuentemente tratados como absolutos- en detrimento de “valores personales” fundamentales como la autonomía y la dignidad^2^.

De forma complementaria, la bioética cotidiana de Giovanni Berlinguer enfatiza la persistencia del sufrimiento social cotidiano, incluso en el proceso de morir, y sostiene que los conflictos éticos relacionados con el vivir y el morir deben ser enfrentados de modo democrático, sin la monopolización por parte de grupos de poder religiosos, políticos o corporativos^13^. Esta perspectiva permite identificar, en los documentos legislativos, dinámicas de poder mediante las cuales fuerzas políticas neoconservadoras operan en la imposición de valores y concepciones particulares de vida y muerte digna, limitando la pluralidad moral necesaria en una sociedad democrática^14^.

Estos marcos teóricos se articulan al reconocer que el lenguaje jurídico-legislativo y los discursos políticos desempeñan un papel central en la traducción (o distorsión) de los valores éticos que orientan la asistencia en salud. Las normas no solo regulan conductas, sino que también producen sentidos morales, delimitan la actuación profesional y moldean las posibilidades de autodeterminación de las personas en situaciones de terminalidad.

En el contexto brasileño, esta discusión se articula directamente con los cuidados paliativos y con las directivas anticipadas de voluntad. La autonomía y el consentimiento informado constituyen pilares éticos tanto en la investigación como en el proceso de atención a la persona al final de la vida^15^. Las directivas anticipadas de voluntad -instrumento mediante el cual la persona expresa su voluntad, e indica qué decisiones deberán tomarse ante el proceso de muerte o designa un responsable para el caso en que no pueda expresarse^13^- se caracteriza como un instrumento fundamental para preservar la autonomía y la dignidad en situaciones de terminalidad, garantizando la autodeterminación^16^^,^^17^ y contribuyendo a evitar conflictos entre familiares y profesionales de la salud en momentos decisorios críticos.

Las políticas públicas, como la Política Nacional de Cuidados Paliativos (PNCP), y los códigos éticos de las profesiones de la salud refuerzan este entendimiento al reconocer la voluntad de la persona como orientadora de las decisiones clínicas en situaciones de enfermedad incurable y terminal. La Política Nacional de Cuidados Paliativos define como cuidados paliativos “las acciones y los servicios de salud destinados al alivio del dolor, del sufrimiento y de otros síntomas en personas que enfrentan enfermedades u otras condiciones de salud que amenazan o limitan la continuidad de la vida” y, entre sus principios orientadores fundamentales, resalta el respeto por los valores y la autonomía del individuo^18^. De modo convergente, los códigos deontológicos de las profesiones de la salud reafirman estos principios, reconociendo las directivas anticipadas de voluntad como un instrumento central para la efectivización de la autonomía^19^^,^^20^.

Sin embargo, persiste una profunda desconexión entre estas directrices del campo de la salud y los discursos y propuestas legislativas analizados en este estudio. Mientras que las ciencias de la salud han consolidado el entendimiento de que las directivas anticipadas de voluntad protegen la autonomía^16^^,^^21^, diversos proyectos legislativos buscan configurarlas como mecanismos de inversión. De modo semejante, aunque los códigos profesionales autorizan la ortotanasia -es decir, la limitación o suspensión de tratamientos fútiles en consonancia con la voluntad del paciente^20^- algunas propuestas legislativas introducen ambigüedades jurídicas que pueden resultar en su criminalización^14^^,^^22^. Es esa tensión -entre lo que las ciencias de la salud reconocen como éticamente adecuado y lo que el Poder Legislativo intenta normativizar- la que fundamenta y justifica la presente investigación.

Desde este marco, el objetivo de este estudio es comprender cómo las prácticas legislativas y políticas del Poder Legislativo brasileño, entre 1981 y 2020, construyeron sentidos, atribuciones y restricciones en torno a las prácticas de fin de vida (eutanasia, suicidio asistido, ortotanasia, cuidados paliativos) y a las directivas anticipadas de voluntad, identificando conflictos, tensiones morales y jerarquías de valores que estructuran estos discursos y su articulación con los principios constitucionales de dignidad humana y autonomía. Ante ello, se optó por una investigación documental cualitativa, basada en la hermenéutica-dialéctica^23^, la cual permite aprehender los discursos legislativos como expresiones de conflictos morales y normativos históricamente situados.

## Metodología

Se trata de una investigación documental cualitativa, orientada por el enfoque hermenéutico-dialéctico, adoptado como un “camino de pensamiento^”(^^23^ capaz de articular el análisis empírico de documentos legislativos con la reflexión crítica sobre valores morales, normas jurídicas y prácticas de salud al final de la vida. La elección de este encuadre metodológico se deriva directamente del objetivo del estudio, ya que la investigación busca comprender cómo los parlamentarios brasileños construyen sentidos éticos, axiológicos y normativos sobre las prácticas de fin de vida y las directivas anticipadas de voluntad.

La hermenéutica-dialéctica, tal como fue sistematizada por Maria Cecília de Souza Minayo, permite aprehender los textos legislativos no solo como enunciados normativos aislados, sino como expresiones de conflictos, tensiones y disputas de valores históricamente situados^23^. Esta perspectiva interpretativa posibilita analizar los discursos del Poder Legislativo Federal brasileño como producciones simbólicas que reflejan y, simultáneamente, configuran concepciones sociales sobre autonomía, dignidad humana, valor de la vida y prácticas de fin de vida. El referente teórico de la bioética de la responsabilidad, de Diego Gracia^2^^,^^3^, y de la bioética cotidiana, de Giovanni Berlinguer^13^, orientó transversalmente el proceso analítico, funcionando como eje interpretativo para la identificación de las jerarquías axiológicas concretas movilizadas en los documentos y para la comprensión de los conflictos ético-normativos presentes en ellos.

El procedimiento analítico siguió las etapas de operacionalización metodológica propuestas por Minayo, adaptadas al objeto y a la pregunta de investigación^23^. En la primera etapa, referida a la elaboración de categorías analíticas, se realizó la búsqueda y selección del conjunto documental. 

Los documentos fueron recolectados en los sitios electrónicos oficiales del Congreso Nacional, de la Cámara de Diputados y del Senado Federal, en los que se realizó una búsqueda utilizando los términos combinados con operadores booleanos: “eutanasia” OR “suicidio asistido” OR “cuidados paliativos” OR “ortotanasia” OR “distanasia” OR “muerte digna”, desde 1981 hasta el 6 de septiembre de 2020. La delimitación temporal (1981-2020) se justifica por el inicio de la disponibilidad de documentos legislativos digitalizados en los repositorios públicos, en 1981, y por el hecho de que, en 2020, los debates normativos sobre terminalidad y prácticas de fin de vida ya se encontraban consolidados en el Poder Legislativo brasileño, con proyectos aún en trámite en el Congreso Nacional.

Como criterio de inclusión, se consideraron textos que trataban explícitamente sobre eutanasia, suicidio asistido, cuidados paliativos, ortotanasia, distanasia, muerte digna, directivas anticipadas de voluntad, testamento vital o mandato duradero, incluyendo propuestas legislativas, sus justificaciones y discursos relacionados con estas prácticas en el contexto del Legislativo federal. Se excluyeron documentos que trataban exclusivamente sobre prácticas veterinarias o sobre cuidados de animales.

El cuerpo documental quedó compuesto por 193 documentos legislativos producidos en el ámbito del Poder Legislativo Federal brasileño, incluidos proyectos de ley sobre eutanasia y ortotanasia (y sus respectivas justificaciones), además de requerimientos, dictámenes y discursos en el plenario.

En la etapa de ordenamiento y clasificación de datos, se realizó inicialmente una lectura exploratoria de los textos, con el fin de familiarizarse con el material e identificar núcleos temáticos recurrentes a partir de las categorías analíticas delineadas en la fase exploratoria. A continuación, los documentos fueron importados al software ATLAS.ti, en el cual se procedió a la codificación temática de los textos, tomando como base unidades de sentido extraídas del acervo. La codificación se realizó de forma empírico-analítica: por un lado, permitió la emergencia de categorías directamente del material empírico; por otro, estuvo orientada por los conceptos teóricos de autonomía, dignidad humana, valores morales, responsabilidad y bioética cotidiana. Los códigos temáticos fueron continuamente comparados, agrupados y refinados, dando origen a categorías analíticas más amplias que expresan patrones argumentativos y normativos presentes en los discursos legislativos, los proyectos de ley y sus justificaciones.

En la etapa siguiente, se procedió a una lectura horizontal y vertical de los textos, de la cual emergieron dos categorías temáticas centrales, alineadas con el objetivo de la investigación: 1) “cuidados paliativos: sentidos y atribuciones”, y 2) “instrumentos y mecanismos de acción para la inversión de las directivas anticipadas de voluntad”. Estas categorías expresan patrones argumentativos que estructuran la construcción legislativa de las prácticas de fin de vida y de las directivas anticipadas de voluntad en el ámbito federal brasileño, así como convergencias, divergencias y contradicciones internas identificadas en el conjunto de documentos analizados.

La etapa final consistió en la interpretación hermenéutico-dialéctica de las categorías construidas, buscando comprender -a la luz de los referentes teóricos movilizados- cómo los proyectos legislativos producen sentidos morales y normativos sobre las prácticas de fin de vida y las directivas anticipadas de voluntad. En esta fase, los fragmentos textuales seleccionados fueron tratados como unidades de sentido y articulados empíricamente con los conceptos del referente bioético adoptado. Las interpretaciones consideraron el contexto histórico y político del conjunto de documentos, así como las tensiones entre el valor de la vida, la autonomía y la dignidad, evidenciando los conflictos ético-normativos presentes en el proceso legislativo y la jerarquía normativa producida.

Los extractos citados a lo largo del análisis fueron identificados por la letra “D”, seguida del número correspondiente al documento en el corpus, con el fin de asegurar la trazabilidad y la transparencia en el proceso interpretativo.

A partir de las categorías analíticas propuestas, se presentan los resultados y se realizan, simultáneamente, las discusiones en las subsecciones que siguen. 

Para este artículo se seleccionaron únicamente dos de las categorías temáticas resultantes de la fase exploratoria de la investigación y de la lectura horizontal/transversal de los documentos^23^, incluidos los proyectos de ley. Esta elección se justifica por el hecho de que el presente texto constituye un recorte de los resultados de la tesis doctoral, alineado con el objetivo específico propuesto para este artículo.

## Resultado y discusiones

### Cuidados paliativos: sentidos y atribuciones

En esta categoría se observa que los cuidados paliativos se introducen en los proyectos legislativos de forma ambigua: aunque reconocidos como práctica legítima en el contexto del final de la vida, sus sentidos son progresivamente restringidos por interpretaciones normativas que limitan su aplicación clínica y ética. Las proposiciones y los discursos parlamentarios tienden a asociar, en el conjunto de los documentos analizados, los cuidados paliativos con la contención del sufrimiento únicamente en la medida en que estos no desafían concepciones morales centradas en la inviolabilidad absoluta de la vida. Fragmentos como el que se presenta a continuación evidencian este movimiento al reconocer los avances tecnológicos y, simultáneamente, expresar temores respecto de la autonomía del paciente en las decisiones sobre tratamientos al final de la vida. Así declara uno de los legisladores:

“En las últimas décadas hemos sido testigos de un gran avance tecnológico en el área de la salud, lo que ha contribuido al prolongamiento de la vida mediante un soporte clínico intensivo. Por un lado, no se puede negar que los avances han traído beneficios para diversas personas con enfermedades graves. Por otro lado, han surgido numerosos cuestionamientos en el campo de la bioética, principalmente en relación con temas como la terminalidad de la vida y la autonomía de las personas para decidir sobre los tratamientos a los que desean someterse, especialmente aquellas con enfermedad en etapa avanzada y sin ninguna perspectiva de cura”. (D40, Justificación del Proyecto de Ley del Senado No. 149 de 2018)

Este enunciado constituye una unidad de sentido que revela el intento de establecer fronteras morales rígidas -entre prolongar la vida y garantizar una muerte digna- fundamentadas en valores vitales jerárquicamente situados por encima de otros valores constitutivos de la persona. Además, denota rasgos de visiones políticas retrógradas que, de manera implícita, buscan restringir avances técnico-científicos en el ámbito de los cuidados paliativos e interferir en las relaciones ético-morales involucradas en estas prácticas, como en los intentos de alterar atribuciones profesionales o criminalizar acciones clínicamente reconocidas para el alivio del sufrimiento, como la sedación paliativa^6^^,^^7^.

Aunque muchas de las propuestas legislativas enfatizan el sufrimiento cotidiano derivado de los avances tecnológicos en los servicios de atención de salud, entre los legisladores predomina un enfoque utilitarista para delimitar parámetros técnicos de intervención médica en el proceso de morir. Esta construcción normativa se apoya en la bioética médica y sostiene que “las conductas médicas restrictivas se basan en criterios médico-científicos de indicación o no indicación de una medida, según su utilidad para el paciente”^24^. El fragmento discursivo que sigue ilustra el énfasis en el sufrimiento provocado por el prolongamiento artificial de la vida, asociando la pérdida del valor de la vida biológica al criterio de utilidad y compasión:

“Establecer límites razonables para la intervención humana en el proceso de morir. La prolongación indefinida de la vida, aunque posible, no siempre será deseable. Es factible mantener las funciones vitales en funcionamiento incluso en casos de precariedad extrema; en ocasiones, incluso en estado vegetativo. Sin embargo, en muchos casos, ese sufrimiento y esa agonía son inhumanos, indignos y atentan contra la propia naturaleza del ciclo de la vida y de la muerte”. (D27, Justificación del Proyecto de Ley No. 3002 de 2008)

El siguiente fragmento también moviliza la compasión y la piedad, anclándose en valores religiosos para admitir la no continuidad de la prolongación artificial de la vida frente a la inevitabilidad de la muerte, evidenciando posturas semejantes basadas en su jerarquía de valores y experiencias personales:

“Soy un hombre extremadamente católico, religioso, pero creo que es una falta de piedad, cuando ya no hay más recursos ni soluciones, prolongar la vida artificialmente. Tuve un caso en mi familia, cuando mi propia madre pasó 11 meses en sufrimiento, conectada a una máquina y sin solución. Eso es falta de piedad. Tenemos que pensar muy seriamente sobre esta situación y debemos decidir con valentía, pero pienso que, artificialmente, no se debe prolongar el sufrimiento de un ser humano”. (D147, discusión parlamentaria del Proyecto de Ley No. 3002 de 2008)

En ambos fragmentos se evidencia que la jerarquización de la dignidad y de la compasión por encima de la vida biológica no se traduce en la defensa del valor de la autonomía de la persona. Los fragmentos revelan que la autodeterminación del sujeto en fase de enfermedad terminal es relegada a un plano secundario: los legisladores admiten que la “prolongación indefinida de la vida” puede no ser “deseable”, pero lo hacen principalmente con base en valoraciones morales, emocionales o religiosas. En este sentido, Gracia^2^ advierte que “las valoraciones no son completamente racionales, pero pueden y deben ser razonables”, siendo la deliberación moral -que también es política- el espacio legítimo para la ponderación de valores en la esfera pública y privada^3^.

Se observa, así, una oposición compasiva al sufrimiento inhumano causado por la obstinación terapéutica (distanasia o futilidad médica), práctica basada en la aplicación de métodos extraordinarios y desproporcionados de soporte vital a pacientes con enfermedades terminales, prolongando su agonía sin beneficio alguno^25^. Su fundamento ético está intrínsecamente ligado al vitalismo médico y al principio de la sacralidad de la vida^26^. Sin embargo, aunque la distanasia sea considerada éticamente inaceptable por violar los principios de la bioética y afrontar la dignidad humana^25^, de los discursos analizados se desprende que en el criterio para no “mantener artificialmente las funciones vitales” no predomina la autonomía o la dignidad, sino la aceptación de la pérdida del valor intrínseco de la vida. De ello se deriva que, en las proposiciones legislativas sobre la ortotanasia, el poder decisorio sobre el cuerpo y el proceso de morir permanece desplazado del sujeto, quien es alienado de su autodeterminación^14^.

Estas construcciones valorativas producen efectos prácticos en la autonomía individual, ya que la voluntad de la persona puede o no ser considerada según criterios técnicos o parámetros de proporcionalidad terapéutica. Tal dinámica puede “perjudicar la autonomía personal, que es precisamente el valor fundamental violado”^13^^)^ por la medicalización de la muerte. De manera similar, la argumentación compasiva sobre el sufrimiento derivado de “las obstinadas intervenciones biotecnológicas”^26^, presente en diversas justificaciones legislativas, se basa en categorías axiológicas inferiores, absolutizando el valor de la vida o su pérdida. Tal movimiento se expresa de manera emblemática en el siguiente fragmento:

“Al garantizar los derechos individuales, fundamentales e inviolables a todas las personas, sin cualquier distinción y, sin distinguir tampoco la etapa de la vida en la que se encuentran, la Constitución Federal cita, en primer lugar, el derecho a la vida. Lo hace con toda lógica, puesto que, sin ese derecho, que es de todos, ningún sentido tendrían los demás”. (D63, Justificación del Proyecto de Ley No. 5058 de 2005)

Además, este segmento evidencia no solo la fuerza de las jerarquías de valores en la constitución moral y normativa de la sociedad, como observa Gracia^2^, sino también el modo en que los valores vitales son interpretados y movilizados, oscureciendo el debate sobre las prácticas de fin de vida y suprimiendo el derecho a morir con dignidad. La interpretación del principio de la “inviolabilidad de la vida” (CRFB, art. 5º) como valor absoluto, supuestamente dotado de precedencia constitucional, ignora que la dignidad humana (CRFB, art. 1º, III) constituye igualmente un pilar fundamental^4^. Así, interpretaciones axiológicas particulares refuerzan la subordinación de la autonomía y de la dignidad a la preservación biológica de la vida, configurando falsos conflictos éticos. Considerando que tanto la vida como la dignidad de la persona humana son pilares constitucionales, resulta evidente que retóricas ideológicas pueden generar falsos dilemas éticos al negar la autonomía individual, comprendida como “aspecto esencial de la dignidad y libertad del ser humano”^27^, perpetuando el paternalismo autoritario en el campo de la salud.

En el ámbito latinoamericano, Dora Porto destaca que la bioética instauró reflexiones acerca de los derechos individuales y colectivos en salud, así como sobre la autonomía, buscando romper con la tradición paternalista de la práctica médica. La autora observa que, actualmente, el debate sobre la autonomía se polariza entre el derecho a recibir y el derecho a rechazar tratamiento^11^. Esta polarización bioética y normativa repercute en varios países. En Argentina, disputas sobre la negativa a tratamientos médicos al final de la vida llegaron al ámbito judicial, culminando en el reconocimiento del derecho de pacientes con enfermedades terminales a rechazar terapias desproporcionadas respecto de las posibilidades de mejora o que prolonguen el sufrimiento^28^. En México, aunque existe respaldo legal para abandonar tratamientos curativos y optar por cuidados paliativos, el desconocimiento sobre las directivas anticipadas de voluntad vuelve poco efectivo este derecho^29^. Una realidad semejante ocurre en Brasil, a pesar de que el derecho a rechazar tratamiento está constitucionalmente garantizado.

Esta polarización bioética repercute en el Poder Legislativo brasileño, como demuestra la justificación del Proyecto de Ley del Senado No. 267/2018^30^, que cuestiona la licitud de la sedación paliativa al final de la vida:

“…en la medicina actual han adquirido particular relevancia los llamados cuidados paliativos. Estos cuidados buscan hacer más soportable el sufrimiento en la fase aguda de la enfermedad y asegurar que el paciente tenga un adecuado acompañamiento humano. En este contexto, entre otras cuestiones, se plantea el problema de la licitud del recurso a los diversos tipos de analgésicos y sedantes para aliviar el dolor del enfermo, cuando ello comporta el riesgo de abreviar su vida”. (D157, Justificación del Proyecto de Ley No. 267 de 2018)

Esta argumentación presenta incongruencias con el conocimiento científico, ya que la sedación paliativa, cuando se aplica correctamente, no acorta la vida: los estudios muestran que pacientes sedados al final de la vida pueden sobrevivir más que los no sedados^31^. Además, refuerza estigmas y tabúes sobre el morir, expresados en una valoración absoluta de la vida desde una perspectiva legislativa neoconservadora, que busca imponer moralmente determinados modos sufrientes de morir mediante normas jurídicas. A lo largo de los proyectos analizados se identifican intentos de redefinir prácticas paliativas, presentándolas como moralmente sospechosas o jurídicamente ambiguas. Estos desplazamientos discursivos configuran proposiciones normativas que pueden debilitar la práctica clínica al imponer restricciones fundamentadas en argumentos morales desprovistos de sustento técnico-científico.

Incluso, cuando no se convierten en normas jurídicas, los discursos político-legislativos pueden producir efectos sociales profundos al cuestionar la legitimidad ética de los cuidados paliativos y subordinar la muerte digna al prolongamiento biológico de la vida. Lo que está en cuestión no es el rechazo a los cuidados paliativos, ampliamente aceptados socialmente -y considerados un “derecho de todos y deber del Estado”^4^, aunque su implementación sea un desafío constante-, sino el derecho incondicional a rechazar tratamientos fútiles. En México, una investigación reciente demostró que, tras recibir información, los participantes prefieren sustituir tratamientos curativos por cuidados paliativos para evitar sufrimientos u obstinación terapéutica frente a una enfermedad terminal^29^. Por lo tanto, el debate legislativo debería centrarse en el derecho a rechazar tratamientos desproporcionados o fútiles, y no en restringir las prácticas paliativas. Sin embargo, la postura legislativa busca limitar el uso de los cuidados paliativos, convirtiendo procedimientos lícitos (como la sedación paliativa) en prácticas potencialmente ilícitas, retirando a la persona el derecho de elegir entre alternativas que respondan a sus necesidades.

Además de estos obstáculos, otros proyectos de ley -como el Proyecto de Ley del Senado No. 6715/2009^32^ y el Proyecto de Ley 6544/2009^33^, sumados al Proyecto de Ley No. 3002/2008^32^, que busca reglamentar la práctica de la ortotanasia- imponen condiciones al ejercicio del derecho a rechazar tratamientos. Tales proyectos establecen que, habiendo una solicitud expresa y por escrito del enfermo en fase terminal de enfermedad o de su representante legal, se permite al médico tratante la práctica de la ortotanasia, definida en estos proyectos como la limitación o la suspensión de procedimientos y tratamientos desproporcionados o extraordinarios. Además, determinan que la decisión relativa a la solicitud del paciente deberá ser emitida por una junta médica especializada (D110 y D112, Cf. Párrafo VII del Artículo 2 del Proyecto de Ley No. 3002 de 2008).

La propuesta subordina claramente el derecho de elección de las personas al poder de decisión corporativo-médico sobre los cuerpos. En defensa de la incondicionalidad del rechazo terapéutico, Lara y Filho afirman que:

“…el derecho al libre desarrollo de la personalidad abarca, como regla, la posibilidad de rechazo terapéutico […] incluso en casos de riesgo inminente de muerte [siendo esta] la decisión más consonante con un Estado democrático de derecho y con los valores y principios positivizados en la Constitución de 1988”.^5^

En síntesis, si por un lado los discursos parlamentarios parecen coherentes al defender la vida, por otro, ocultan concepciones particulares de mundo que se pretende imponer a la sociedad mediante la limitación de prácticas de fin de vida éticamente aceptadas. Como recuerda Giovanni Berlinguer, el derecho a la autonomía del cuerpo debe ser protegido, sobre todo, contra la arbitrariedad del poder político y, “…en lo que respecta a la autonomía personal, entre las decisiones sobre el propio destino, debe incluirse también, de modo laico, la de poder elegir entre continuar o no siendo curado, si vivir o morir”^13^.

El análisis comparativo demuestra que, aunque los parlamentarios invoquen la dignidad y la compasión, tales referencias son, en general, subsumidas por argumentos que absolutizan el derecho a la vida, subordinando la autonomía individual a valores axiológicamente inferiores. El acervo documental revela que las intenciones políticas expresadas en los proyectos analizados buscan, bajo un sesgo fundamentalista,

“…imponer sus intereses en detrimento de los intereses nacionales con la ayuda de “brechas” en nuestro ordenamiento jurídico, que debe procurar evolucionar, en este caso, para que no se permita el avance de los movimientos de relativización de la vida”. (D75, Justificación del Proyecto de Ley N° 580 de 2020)

Se verificó que el debate es monopolizado por fuerzas conservadoras hegemónicas cuyos intereses políticos tienden a hacer retroceder el desarrollo moral e histórico, especialmente en el campo de la salud.

### Instrumentos y mecanismos de acción para la inversión de las directivas anticipadas de voluntad

La segunda categoría evidencia cómo las directivas anticipadas de voluntad son incorporadas al debate legislativo de forma tensionada y, con frecuencia, contradictoria. En la contemporaneidad, conforme señala Giovanni Berlinguer, entre las cuestiones bioéticas cotidianas -es decir, aquellas que ocurren diariamente y que ya no deberían persistir- se encuentra la muerte con sufrimiento de personas al final de la vida^13^. Las directivas anticipadas de voluntad surgen, en este contexto, como un instrumento orientado a la prevención del sufrimiento evitable. Aunque formalmente reconocidas como expresiones de la autonomía, numerosas propuestas legislativas buscan someterlas a condicionamientos legales y procedimentales que vacían su potencial emancipador, configurando una injerencia estatal e institucional en la vida privada, así como un dominio sobre el otro en el proceso de toma de decisiones al final de la vida.

Tales propuestas confrontan construcciones éticas y científicas consolidadas, desarrolladas en el ámbito académico y en las organizaciones profesionales, que resultaron en normas infralegales que actualmente aseguran la legitimidad institucional de las directivas anticipadas de voluntad. En este sentido, las directivas anticipadas de voluntad, conforme se describe en la Resolución No. 41, del 31 de octubre de 2018, del Ministerio de Salud de Brasil, es definida como uno de los “principios orientadores para la organización de los cuidados paliativos”^34^. De manera convergente, la Política Nacional de Cuidados Paliativos establece, entre sus directrices, la obligatoriedad de la “observancia de la Directiva Anticipada de Voluntad de la persona atendida”^18^. En cuanto a su forma, las directivas anticipadas de voluntad se caracterizan como:

“…el documento de instrucciones previas de voluntad es un documento sanitario de carácter declarativo, particular, que deberá ser elaborado por escrito, firmado por el autor, en presencia de dos testigos, todos en pleno goce de sus capacidades civiles y cognitivas, debiendo las firmas ser reconocidas conforme a la ley, y el documento deberá identificar civilmente al autor y a los dos testigos”. (D40, Artículo 30 del Proyecto de Ley No. 352 de 2019)

Sin embargo, la insuficiente difusión de información y el desconocimiento de la población acerca del derecho a las directivas anticipadas de voluntad hacen que este instrumento -potencialmente capaz de evitar la obstinación terapéutica- sea poco accesible en la práctica. Se trata de un problema recurrente en diversos países latinoamericanos. En México, por ejemplo, una investigación realizada mediante un cuestionario aplicado a 108 adultos, mayoritariamente personas mayores, en una región metropolitana, “revela desconocimiento sobre las directivas anticipadas de voluntad: su forma, contenido y alcance”^29^.

En Brasil, frente a la indefinición normativa respecto del contenido, el alcance y los límites de las directivas anticipadas de voluntad, tramitan proyectos de ley con perspectivas distintas y, en ocasiones, conflictivas. El Proyecto de Ley del Senado 149/2018, que “dispone sobre las directivas anticipadas de voluntad sobre tratamientos de salud”^35^, se fundamenta en una justificación alineada con lo que ya se encuentra éticamente establecido en diversos países:

“Numerosos países cuentan con legislación de este tipo, como los Estados Unidos de América, Argentina y diversos países de la Comunidad Europea, como España, Italia, Portugal, Suiza y Holanda. Así, es necesario colocar a Brasil en consonancia con la tendencia mundial de garantizar, mediante una ley, la posibilidad de que el paciente manifieste y tenga respetada su voluntad antes de la aparición o del agravamiento de una enfermedad grave, indicando expresamente a qué tratamientos acepta o rechaza someterse o, incluso, nombrando a un representante para decidir por él en caso de volverse incapaz”. (D148, Justificación del Proyecto de Ley No. PL 6715 de 2009)

Este proyecto retoma, como señala Dora Porto, la centralidad del debate bioético en torno al derecho a recibir o rechazar tratamiento, configurándose, a primera vista, como una propuesta garantista, alineada con lo que ya es constitucionalmente aceptado^11^. De hecho, un estudio sobre “la falta de legislación que proteja el derecho de los pacientes […] en Brasil” concluye que, constitucionalmente, “es absoluto el derecho de los pacientes capaces de elegir tratamiento médico en armonía con sus valores personales”^36^. El derecho a la privacidad, consagrado constitucionalmente, garantiza al paciente la posibilidad de decidir sobre su tratamiento médico, incluso de aceptar o rechazar intervenciones, aun cuando ese rechazo resulte en la muerte^36^. No obstante, los proyectos legislativos, en el corpus examinado, restringen las directivas anticipadas a la “persona en fase terminal de enfermedad o afectada por daños graves e irreversibles a la salud”, además de prohibir, de forma genérica, “la negativa a tratamientos paliativos”^35^.

No tardaron en surgir dictámenes con manifestaciones aún más restrictivas, marcadas por tensiones e intereses corporativos. Un dictamen de la Comisión de Asuntos Sociales, relativo al Proyecto de Ley del Senado 149/2018^35^, afirma que:

“…para que la declaración de voluntad del paciente sea reconocida por los profesionales y por los servicios de salud, el documento deberá estar expresado en escritura pública sin contenido financiero, otorgada ante el registro notarial competente. Además, únicamente los cuidados que tengan por finalidad retardar el proceso natural de muerte podrán ser objeto de disposiciones sobre interrupción de tratamiento en las directivas anticipadas de voluntad, prohibiéndose la negativa a tratamientos paliativos. De esta forma, creemos que quedan asegurados el respeto a la autodeterminación de la persona -en consonancia con el principio de la dignidad humana, tutelado constitucionalmente como principio fundamental del orden jurídico brasileño- y los cuidados básicos indispensables para garantizar una muerte digna al paciente”. (D150, Dictamen técnico de la Comisión de Asuntos Sociales sobre el Proyecto de Ley del Senado No. 149 de 2018)

Las contradicciones en la formulación de criterios que limitan la declaración de voluntad del individuo se vuelven aún más evidentes con el Proyecto de Ley del Senado 267/2018, que amplía las restricciones al derecho de rechazar procedimientos y tratamientos médicos. Conforme a lo dispuesto en su art. 3º:

“La manifestación de voluntad del declarante, al elaborar sus directivas anticipadas de voluntad, deberá explicitar los cuidados, tratamientos y procedimientos que acepta, siéndole, sin embargo, vedado: I) rechazar cuidados paliativos, en particular en lo relativo al control de síntomas; II) realizar solicitud de muerte asistida; III) realizar disposiciones de carácter patrimonial; IV) manifestarse acerca de la autocuratela y de la toma de decisión asistida. §1º En el ámbito de las directivas anticipadas de voluntad, el declarante podrá rechazar cuidados, tratamientos y procedimientos de salud que tengan como objetivo prolongar su vida biológica…”.^30^

Se observa, así, que los legisladores, a partir de sus propias jerarquías axiológicas, procuran imponer condicionamientos valorativos particulares sobre la vida y la muerte de toda la colectividad. En este proceso, los cuidados paliativos son movilizados tanto como sustitutos de la garantía constitucional de no ser sometido a tratamientos médicos contra la propia voluntad como alternativa discursiva a las proposiciones sobre eutanasia. Tal estrategia aparece en una enmienda al Proyecto de Ley del Senado 236/2012, que propone la “reforma del Código Penal Brasileño”^37^:

“En realidad, la materia relativa a los pacientes en estado terminal de enfermedad ya viene siendo debatida en el Congreso Nacional desde hace bastante tiempo, incluso con la realización de audiencias públicas. De ello resulta el perfeccionamiento de proyectos de ley que han sido aprobados, tanto en el Senado como en la Cámara, apartando la práctica de la eutanasia y acogiendo y regulando los cuidados paliativos que deberán ser siempre garantizados, incluso en respeto a los derechos constitucionales de la vida, de la salud y de la dignidad de la persona humana”. (D151, Justificación de la modificación del artículo 126 del Proyecto de Ley No. 236 de 2012)

En esta lógica, las directivas anticipadas de voluntad son reconfiguradas normativamente y dejan de operar como instrumentos de garantía de la autonomía individual para asumir la función de mecanismos de poder médico-estatal y de interferencia moral e institucional sobre los cuerpos. Tal inversión, no solo vulnera el principio constitucional de la dignidad humana, sino que también representa un retroceso respecto del progreso moral que, en teoría, habría superado el paternalismo autoritario. En consecuencia, contradice la “teoría del consentimiento informado”, según la cual “…en principio, y salvo excepciones, nadie puede actuar sobre el cuerpo de otra persona sin informarla de ello y recibir de ella autorización. Lo demás es agresión física pura y simple, aun cuando el objetivo sea aquello que el profesional considere beneficioso para la persona”^1^.

La subordinación de la autonomía al poder médico se extiende también a los tipos de directivas anticipadas de voluntad denominadas “testamento vital” -también sometidas a limitaciones semejantes, sobre todo por su subordinación a la lógica de medicalización de la vida-, así como al “mandato duradero”, mediante el cual la persona nombra “…uno o más apoderados que deberán ser consultados por los médicos en caso de [su] incapacidad, definitiva o no, cuando estos deban tomar alguna decisión sobre el rechazo de tratamiento”^16^. No obstante, como observa la propia autora, para que las disposiciones de rechazo o aceptación de cuidados y tratamientos sean válidas en el ordenamiento jurídico brasileño, “el paciente no podrá disponer acerca del rechazo de los cuidados paliativos”^21^, tal como prevén los proyectos de ley analizados.

En el conjunto documental examinado se identifica un patrón recurrente de inversión del sentido original de las directivas anticipadas de voluntad: en lugar de funcionar como instrumentos de autodeterminación, los discursos y proposiciones legislativas proponen que operen como mecanismos de control externo sobre las decisiones de la persona al final de la vida. La comparación entre los proyectos revela variaciones temporales y argumentativas, pero converge en un núcleo común: el mantenimiento de amplios márgenes de intervención médica, institucional (incluso familiar) sobre la voluntad previamente expresada por el individuo. Esta convergencia refleja una jerarquización axiológica en la que prevalecen valores morales de baja jerarquía, especialmente valores vitales, en detrimento de la autonomía y de la dignidad humana.

Las contradicciones internas del discurso legislativo se vuelven especialmente evidentes cuando, simultáneamente, se afirma la protección de la dignidad de la persona humana y se instituyen dispositivos que limitan drásticamente su capacidad de decisión autónoma. Esta tensión revela un conflicto ético-normativo estructural en la construcción legislativa de las prácticas de fin de vida.

En síntesis, las disputas políticas se muestran inequívocas. La base de estos conflictos reside en la paradoja de las interpretaciones de los principios constitucionales, especialmente en lo que se refiere a la inviolabilidad de la vida y a la dignidad humana. Con frecuencia, las posiciones contrarias a cualquier avance que amplíe la libertad decisoria asumen la inviolabilidad de la vida humana como absoluta, situándola en la cima de la jerarquía axiológica, independientemente del contexto y de las consecuencias para la dignidad humana. De este modo, el derecho a la vida se disocia del derecho a morir con dignidad, en contraste, por ejemplo, con interpretaciones jurídicas como la que fundamentó la despenalización de la eutanasia en Colombia, según la cual el derecho a una vida digna implica, necesariamente, el derecho a morir con dignidad^38^. Se deduce, así, que el valor de la vida ante la muerte no puede ser considerado más relevante que la libertad, la autonomía y la dignidad humana. En el proceso de “deliberación ética”, es necesario ponderar “todos los valores -tanto los públicos como los privados”^2^-, así como los proyectos de vida de cada persona. La deliberación ética debe constituir la base de la deliberación política. Como afirma Diego Gracia: “la deliberación política no puede ser sino un epifenómeno de la deliberación moral […]. Dime qué valores tienes y te diré cuáles serán tus leyes”^2^.

## Consideraciones finales

Este estudio analizó cómo los discursos y las proposiciones del Poder Legislativo Federal brasileño, entre 1981 y 2020, construyen sentidos morales y normativos acerca de las prácticas de fin de vida -especialmente los cuidados paliativos- y de las directivas anticipadas de voluntad, evidenciando disputas axiológicas, tensiones morales y jerarquías de valores que inciden sobre la autonomía, la dignidad humana y la responsabilidad moral en el proceso de morir. Se partió del reconocimiento de una contradicción normativa central: aunque la dignidad de la persona humana y la autonomía individual constituyen principios constitucionales fundamentales, los debates legislativos analizados tienden, de manera predominante, a absolutizar el valor de la vida biológica en detrimento de estas dimensiones constitutivas de la persona, oponiendo vida y dignidad como si fueran excluyentes.

El análisis hermenéutico-dialéctico -articulado con los referentes de la bioética de la responsabilidad de Diego Gracia y de la bioética cotidiana de Giovanni Berlinguer^1^^,^^2^^,^^13^- evidenció cómo valores vitales y concepciones moralmente conservadoras configuran la formulación normativa del morir, muchas veces en desacuerdo con marcos éticos contemporáneos, políticas públicas de cuidados paliativos y garantías constitucionales. En consecuencia, tales discursos producen sentidos morales que orientan y, en diversos casos, restringen las posibilidades de autodeterminación al final de la vida.

Los resultados revelaron dos movimientos centrales. En la primera categoría temática -*cuidados paliativos: sentidos y atribuciones*- se identificó que los cuidados paliativos son movilizados de forma ambigua: a pesar de ser reconocidos como prácticas legítimas para promover el derecho a morir con dignidad, sus sentidos son limitados por interpretaciones normativas que los subordinan a concepciones moralizantes centradas en la inviolabilidad absoluta de la vida. Desde esta perspectiva, el derecho a rechazar tratamientos fútiles o desproporcionados es, en la mayoría de los casos, negado, desplazando el eje decisorio del sujeto hacia instancias médicas, institucionales o jurídicas.

Además, se constató que los cuidados paliativos son recurrentemente reinterpretados en el debate legislativo a partir de criterios utilitaristas, moralizantes o religiosos, siendo en ocasiones presentados como la única alternativa frente a prácticas de fin de vida criminalizadas, como la eutanasia y el suicidio asistido. En estas construcciones, prácticas clínicamente consolidadas -como la limitación o suspensión de tratamientos fútiles (ortotanasia) y la sedación paliativa- pasan a ser tratadas como jurídicamente sospechosas o moralmente ambiguas, poniendo en duda su legitimidad ética. Aunque los parlamentarios reconozcan el sufrimiento asociado a la obstinación terapéutica, tales reconocimientos rara vez se traducen en la defensa de la autonomía del paciente; por el contrario, sus discursos sostienen la preservación incondicional de la vida biológica, incluso cuando esta carece de un beneficio real para el sujeto.

En la segunda categoría -instrumentos y mecanismos de acción para la inversión de las directivas anticipadas de voluntad- se identificó un patrón recurrente de inversión del sentido de las directivas anticipadas de voluntad. Aunque concebidas para proteger la autonomía, las directivas anticipadas de voluntad pasan a operar, en los proyectos analizados, como mecanismos de control moral e institucional. Las proposiciones legislativas introducen exigencias procedimentales excesivas, restringen contenidos legítimos, condicionan el derecho a rechazar tratamientos y prohíben, de forma genérica, la negativa a cuidados paliativos, reforzando prácticas autoritarias ampliamente criticadas en los campos clínico y bioético. En ambas categorías, la autonomía aparece relativizada y, en algunos casos, anulada, revelando un patrón legislativo que tiende a reforzar perspectivas paternalistas.

Esta dinámica evidencia una jerarquización axiológica presente en el corpus, en la cual prevalecen valores vitales -situados en los niveles inferiores de la jerarquía, según el referencial axiológico de Diego Gracia^2^- en detrimento de valores personales, como la autonomía y la libertad. Al responder a la pregunta de investigación, el estudio demuestra que los sentidos morales y normativos producidos por el Poder Legislativo brasileño reflejan disputas ético-políticas profundas, en las que se proyectan concepciones particulares de vida y muerte como universales, mediante la normativización jurídica, aunque no todo el Poder Legislativo brasileño opere de esta manera. Estas construcciones disocian el derecho a la vida del derecho a morir con dignidad, generando conflictos ético-normativos que pueden afectar las prácticas institucionales de salud y la experiencia concreta de las personas en situación de terminalidad.

En síntesis, el análisis revela que la construcción legislativa de las prácticas de fin de vida en Brasil permanece marcada por tensiones éticas estructurales, en las cuales los valores vitales frecuentemente se imponen sobre la autonomía y la dignidad humana. Al explicitar estas contradicciones y sus bases axiológicas, sin pretender ofrecer respuestas definitivas, se amplió la comprensión sobre las condiciones que posibilitan, o bloquean, el ejercicio del derecho a morir con dignidad, señalando la necesidad de deliberaciones públicas sensibles a la pluralidad moral, al conocimiento técnico-científico y al reconocimiento de la autonomía como dimensión indisociable de la dignidad humana.
